# Serum Prolidase Activity and Oxidative Stress in Diabetic Nephropathy and End Stage Renal Disease: A Correlative Study with Glucose and Creatinine

**DOI:** 10.1155/2014/291458

**Published:** 2014-09-08

**Authors:** Akhilesh Kumar Verma, Subhash Chandra, Rana Gopal Singh, Tej Bali Singh, Shalabh Srivastava, Ragini Srivastava

**Affiliations:** ^1^Department of Biochemistry, Institute of Medical Sciences, Banaras Hindu University, Varanasi, Uttar Pradesh 221005, India; ^2^Department of Nephrology, Institute of Medical Sciences, Banaras Hindu University, Varanasi, Uttar Pradesh 221005, India; ^3^Division of Biostatistics, Department of Community Medicine, Institute of Medical Sciences, Banaras Hindu University, Varanasi, Uttar Pradesh 221005, India; ^4^Department of Oral Pathology, Jaipur Dental College, Jaipur, Rajasthan 302020, India

## Abstract

Association of oxidative stress and serum prolidase activity (SPA) has been reported in many chronic diseases. The study was aimed at evaluating the correlation of glucose and creatinine to SPA and oxidative stress in patients with diabetic nephropathy (DN) and end stage renal disease (ESRD) concerned with T2DM. 50 healthy volunteers, 50 patients with T2DM, 86 patients with DN, and 43 patients with ESRD were considered as control-1, control-2, case-1, and case-2, respectively. Blood glucose, creatinine, SPA, total oxidant status (TOS), total antioxidant status (TAS), and oxidative stress index (OSI) were measured by colorimetric tests. SPA, TOS, and OSI were significantly increased in case-1 and case-2 than control-1 and control-2, while TAS was significantly decreased (*P* < 0.001). Blood glucose was linearly correlated to SPA, TOS, TAS, and OSI in control-2, case-1 and case-2 (*P* < 0.001). Serum creatinine was linearly correlated with SPA, TOS, TAS and OSI in control-2 and case-1 (*P* < 0.001). In case-2, serum creatinine was significantly correlated with SPA only (*P* < 0.001). Thus, the study concluded that SPA and oxidative stress significantly correlated with blood glucose and creatinine. SPA, TOS, TAS, and OSI can be used as biomarkers for diagnosis of kidney damage.

## 1. Introduction

Chronic kidney disease is one of the major health related issues of twenty-first century. Nephropathy constitutes a major microvascular complication of long term diabetes mellitus and it is the most common cause of end stage renal disease. It is categorized into five different stages. In stage one, the kidney function is normal and in stage two it is slightly reduced. Third and fourth stages are considered as incipient and overt diabetic nephropathy, respectively, while the last or fifth stage of diabetic renal disease is considered as end stage renal disease [1, 2]. Hyperglycemia leads to altered level of non enzymatic protein glycation and glucotoxicity, activation of protein kinase C and increased activity of aldose reductase. These events enhance the production of thromboxane, growth factors, fibronectin and collagen type-IV as well as oxygen free radicals which induces the oxidative stress, resulting in the development and progression of complications of diabetes [3–5].

Prolidase (EC 3.4.13.9) activity is reported as a marker for oxidative stress for many diseases, like diabetes, diabetic neuropathy, nonulcer dyspepsia, chronic liver diseases, erectile dysfunction, osteoporosis, and so forth [6–11]. It cuts dipeptides with proline or hydroxyproline at C-terminal amino end [12, 13] and is involved in cell growth, collagen metabolism, and matrix remodeling [14]. Prolidase activity has been reported in plasma; leukocytes; erythrocytes; in various organs such as kidney and uterus; dermal fibroblasts; and thymus [7]. Very few studies on T2DM have been reported which are controversial with respect to serum prolidase activity [6, 9]. However no one has reported on serum prolidase activity in ESRD with T2DM.

Thus, in the present study, we aimed to observe the correlation of blood glucose and creatinine to serum prolidase activity and oxidative stress in T2DM, diabetic nephropathy and in ESRD with T2DM, and assess the possibility of a new biomarker for the evaluation of kidney damage.

## 2. Subjects and Methods

This study was done at the Department of Biochemistry and Department of Nephrology, Institute of Medical Sciences, Banaras Hindu University, Varanasi, India. The study was ethically approved by institutional ethical committee and signed informed consent was taken from every subject.

### 2.1. Patients Selection

Total of 229 subjects of matched age and sex were selected. Out of 229, 50 subjects (F-16, M-34) who were patients of type-2 diabetes mellitus (T2DM) of ages between 25 and 76 (53.34 ± 14.68) years, 86 patients with diabetic nephropathy (F-39, M-47) of age range 28–76 (52.34 ± 12.89) years, 43 ESRD patients (F-19, M-24) caused by T2DM of age range 30−80 (57.34 ± 15.36) years old, and 50 healthy volunteers (F-20, M-30) of age 25–75 (52.64 ± 18.76) years old were included in the study. Healthy volunteers and patients with T2DM were considered as control-1 and control-2 group, respectively, whereas patients with diabetic nephropathy and ESRD were categorized as case-1 and case-2, respectively (Table 1).

*Inclusion Criteria.* Patients of T2DM, diabetic nephropathy, and ESRD attending outpatient department and admitted in the ward of nephrology, Sir Sunder Lal hospital, BHU, who agreed to participate in the study were included. Medical history, standard physical examination, and test of biochemical parameters (blood sugar, creatinine, and glomerular filtration rate) listed in Table 1 are used for selection and categorization of cases and controls.

*Exclusion Criteria.* Patients suffering from major infections like tuberculosis, HIV, and so forth, chronic heart failure, pregnant females, taking potent antioxidant, and history of alcohol intake were excluded from our study. Diabetic macroangiopathic complications (like coronary artery disease, peripheral vascular disease, and stroke) and acute myocardial infarction in diabetes groups were excluded.

### 2.2. Collection and Storage of Samples

Venous blood was collected in 2 vials (EDTA tube and plane tube). Blood in the EDTA tube was used for the estimation of glucose and hemoglobin, while blood in plane tube was allowed to clot and serum was separated by centrifugation at 3000 rpm for 10 min at 4°C. Serum samples for the measurement of serum prolidase activity, total oxidant status (TOS), and total antioxidant status (TAS) were stored at −80°C. Samples were thawed to room temperature before every assay, and repeated thaw was avoided.

### 2.3. Estimation of Serum Prolidase Activity

Diluting solution (contains 1 mM MnCl_2_ in 6 mM tris HCl buffer), standard proline solution (650 *μ*mol/L in 0.45 mol/L trichloroacetic acid), 94 mmol/L glycyl-l-proline solution (94 mmol/L Gly-l-Pro solution in 0.05 mol/L Tris HCl buffer containing 1 mmol/L of MnCl_2_ (pH 7.8–8.0)), and Chinard's reagent (600 mL of glacial acetic acid mixed with 400 mL of 6 mol/L orthophosphoric acid and dissolved 25 g of ninhydrin in the mixture at 70°C temperature; for preparation of 6 mol/L orthophosphoric acid, 407 mL of orthophosphoric acid (85%, *d* = 1.7) was added to 593 mL of distilled water) were prepared.

## 3. Procedures

The serum obtained from subjects were diluted six times with diluting solution and preincubated for 24 hours at 37°C for enzyme activation. The enzymatic reactions were carried out in two Eppendorf tubes and labeled as experimental and control. 100 *μ*L of Gly-l-Pro and 100 *μ*L of diluted preincubated serum were added in experimental tube, while control tubes contain only 100 *μ*L of diluted preincubated serum. Both tubes were incubated for 30 minutes at 37°C. By adding of 1 mL of trichloroacetic acid (0.45 mol/L) the reaction was checked. Followed by 100 *μ*L of diluted, nonincubated serum was added in control tube. After that centrifugation at 2000 rpm for 5 minutes supernatant was separated and 0.5 mL was used for proline estimation.

For measurement of enzymatic reaction, four tubes were selected and labeled as blank tube, standard tube, experimental tube, and control tube. 1 mL of glacial acetic acid and 1 mL of Chinard's reagent were added in each tube. After this, 0.5 mL of supernatant was added in experimental tube and control tube. 0.5 mL of trichloroacetic acid (0.45 mol/L) was added in blank tube and 0.5 mL of standard proline solution was added in standard tube. All four tubes were incubated in water bath at 90°C for 10 minutes and optical densities (OD) were taken at 515 nm. Spectrophotometer was adjusted to zero with blank tube solution.

For the calculation of enzymatic activity we used the following Myara et al. [8] equation:

(1) E−CS×[S]×2.4=mmol·Min⁡−1·L−1  at  37°C,pH  7.8–8.0,

where *S*: standard tube absorbance, *E*: experimental tube absorbance, *C*: control tube absorbance, and [*S*]: substrate concentration in mmol/L (94 mmol/L).

### 3.1. Total Antioxidant Status (TAS) and Total Oxidant Status (TOS)

TAS and TOS of serum were determined by using automated measurement methods developed by Erel [15, 16].

### 3.2. Oxidative Stress Index (OSI)

OSI values were calculated according to the following formula [11]:

(2) $$\begin{array}{c} \text{OSI}\left(\text{A}\right)=\frac{\text{TOS}\;\;\left(\text{mmol}\;{\text{H}}_{2}{\text{O}}_{2}\;\text{Eq}./\text{L}\right)\;}{\text{TAS}\;\;\left(\mu \text{mol}\;\text{Trolox}\;\text{Eq}./\text{L}\right)}. \end{array}$$

### 3.3. Glomerular Filtration Rate (GFR) Estimation

GFR was calculated according to following CKD-EPI creatinine equation [17]:

(3) Estimated  GFR  (mL/min⁡/1.73 m2) =175×Scr−1.154  ×age−0.203×1.212  [if  black]  ×0.742  [if  female].

This equation was originally designed to estimate glomerular filtration rate and expressed in mL/min/1.73 m^2^. *S*_cr_ = serum creatinine in mg/dL and age in years were used in the equation.

Estimation of clinical parameters like creatinine, blood glucose (fasting blood sugar (FBS), and two hours postprandial blood sugar (PPBS)) and urea were done by commercial kits (creatinine by alkaline picrate method, blood glucose by glucose oxidase method, and urea by DAM method). Hemoglobin was estimated by hematology autoanalyser and blood pressure was measured by mercury sphygmomanometer.

### 3.4. Statistical Analysis

Parametric and nonparametric statistical methods, Student's *t*-test, ANOVA Student-Newman-Keuls test, and Pearson's correlation were used. *P* value less than 0.05, 0.01, and 0.001 were considered as significant, highly significant, and very highly significant, respectively. Sensitivity, specificity, and all other statistical calculations were done by use of software SPSS 16.0 version. Inter- and intraassay % of coefficient of variations (CV) was estimated for SPA (6.85 and 3.82%), TAS (8.45 and 4.12%), and TOS (7.38 and 3.89%).

## 4. Results

### 4.1. Demography and Clinical Parameters for Controls and Cases

Demography and clinical parameters of controls and cases are tabulated in Table 1. One way ANOVA for glomerular filtration rate, serum creatinine (*S*_cr_), fasting blood glucose (FBS), postprandial blood glucose (PPBS), systolic blood pressure, diastolic blood pressure, haemoglobin, and blood urea within and between groups (control-1, control-2, case-1, and case-2) were significant (all *P* < 0.001; Table 1).

### 4.2. Serum Prolidase Activity (SPA) in Controls and Cases

SPA in control-1, control-2, case-1, and case-2 was observed as 55.72 ± 7.90, 60.18 ± 7.85, 68.56 ± 11.09, and 74.23 ± 13.22 mmol min^−1^ L^−1^, respectively (Table 2). Thus SPA is significantly increased in case-2 than in case-1, control-1, and control-2 (*P* < 0.001; Table 2). Differences of SPA between any two groups have significant values (all *P* < 0.05; Table 3).

### 4.3. Total Antioxidant Status (TAS) in Controls and Cases

TAS in case-2 (1.20 ± 0.45 mmol Trolox Eq/L) was significantly decreased than in case-1, control-2 and control-1 (1.42 ± 0.39, 1.82 ± 0.43, and 1.97 ± 0.51 mmol Trolox Eq/L, resp.) (*P* < 0.001; Table 2). TAS was nonsignificantly decreased in control-2 than in control-1 (*P* = 0.091), while differences of TAS for any other two groups were of significant value (all *P* < 0.01; Table 3).

### 4.4. Total Oxidant Status (TOS) in Controls and Cases

TOS in case-2 (22.21 ± 3.66 *μ*mol H_2_O_2_ Eq./L) was significantly increased than in case-1, control-2, and control-1 (20.13 ± 3.75, 17.54 ± 2.46, and 13.82 ± 2.14 *μ*mol H_2_O_2_ Eq./L, resp.) (*P* < 0.001; Table 2). The differences of TOS for any two groups were of significant value (*P* < 0.001; Table 3).

### 4.5. Oxidative Stress Index (OSI) in Controls and Cases

OSI in case-2 (22.09 ± 10.21) was significantly increased than in case-1 (16.04 ± 7.58), control-1 (8.02 ± 4.21), and control-2 (10.58 ± 4.58) (*P* < 0.001; Table 2). OSI in control-2 was nonsignificantly increased than in control-1 (*P* = 0.07, Table 3), while differences in OSI for any other two groups were significant (all *P* < 0.001; Table 3).

### 4.6. Correlative Values

Correlative values of fasting blood sugar (FBS) and creatinine (Cr) with SPA, TOS, TAS, and OSI are tabulated in Table 4. Nonsignificant correlation was observed between FBS and SPA and TOS, and TAS, and OSI in control-1, while significant correlation was observed in control-2, case-1, and case-2. Creatinine was nonsignificantly correlated to SPA, TOS, TAS, and OSI in control-1, while significant correlation was observed in control-2 and case-1 (Table 4). Increased creatinine in case-2 was positively correlated to SPA (*P* < 0.001), while nonsignificant correlation was observed between creatinine and TOS and TAS and OSI (Table 4).

### 4.7. Sensitivity and Specificity of SPA, TOS, TAS, and OSI

**Figure 1:**
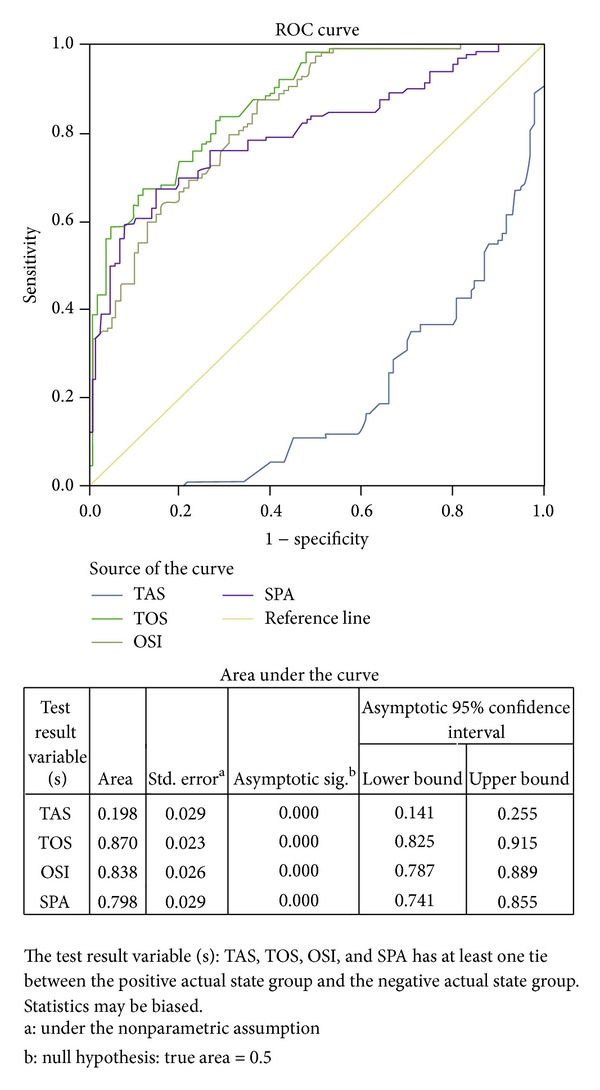
Receiver operating characteristic (ROC) curve of SPA, TOS, TAS, and OSI at cutoff value ≥60.78 mmol min^−1^ L^−1^, ≥16.64 *μ*mol H_2_O_2_ Eq/L, ≤0.81 mmol Trolox Eq/L, and ≥10.24 AU, respectively, for the prediction of kidney damage (either DN or ESRD or both).

DN and ESRD both are different stages of kidney damage. Thus for prediction of kidney damage values of area under the receiver operating characteristic curve (AUROC), sensitivity, and specificity for SPA, TOS, TAS, and OSI were tabulated in Table 5 and Figure 1.

## 5. Discussion

Out of the 229 patients enrolled for the study, 94 were females and 135 were males. As shown in Table 1, mean age group of control-1, control-2, and case-1 is nearly the same, while the mean age for the group case-2 is slightly higher. With increase in the duration of the disease, decline in kidney function (↓ in GFR and haemoglobin and ↑ in serum creatinine and urea along with elevated blood pressure) is clearly seen and it is statistically significant also.

Serum prolidase activity, TOS, TAS, and OSI are recognized as oxidative stress markers. In our study, patients with ESRD have increased SPA, TOS, and OSI and decreased TAS than patients with DN, T2DM, and healthy volunteers (*P* < 0.001) (Table 2). Patients with T2DM and DN also have increased oxidative stress than healthy volunteers and the increase is also seen with the progression of the disease.

Erbagci et al, [9], have reported that serum prolidase activity was decreased in patients with T2DM than healthy volunteers. On contrary, experimental results of Uzar et al [6], showed that serum prolidase activity was increased with increase in oxidative stress in patients with diabetes than normal subjects. Our present study shows that increase in serum prolidase activity and TOS were significant (all *P* < 0.05) and increase in OSI was non-significant (*P* = 0.07), while TAS was non-significantly decreased (*P* = 0.091) in patients with T2DM than healthy volunteers (Table 3). Erbagci et al. [9], has reported that differences in prolidase activity between with and without diabetic nephropathy were non-significant. In present study, we found significant increase in serum prolidase activity, TOS, OSI and significant decrease in TAS in patients with DN and ESRD than healthy volunteers (all *P* < 0.001, Table 3).

In patients with T2DM and DN, oxygen free radicals increase with continuous elevation of glucose and this leads to increased oxidative stress [18, 19]. In our present study we observed that mean of duration of diabetes continuously increases as the disease progresses from T2DM to DN to ESRD (Table 1). This increase in duration of diabetes positively correlates with increase in TOS and OSI (all *P* < 0.05), while increase in duration of diabetes negatively correlates with decrease in TAS (all *P* < 0.01; Table 4). Observed SPA strongly positively correlated to duration of diabetes in patients with T2DM, DN, and ESRD (all *P* < 0.001; Table 4). Thus it seems that with increase in duration of the disease, continuous increase in oxidative stress is observed and that might be the one mechanism for pathogenesis of progression of the disease.

Chronic high blood glucose leads to glucose toxicity, which is responsible for chronic oxidative stress in patients with T2DM [20, 21]. In our previous studies, we showed that increased oxidative stress is related to increased inflammatory cytokines,* Helicobacter* infection, and bacterial meningitis [22, 23]. In present study it is shown that SPA, TOS, and OSI were significantly increased and TAS was significantly decreased in patients with T2DM, DN, and ESRD than healthy subjects. This increase in SPA, TOS, and OSI and decrease in TAS were significantly correlated to chronic high blood glucose in T2DM, DN, and ESRD (all *P* < 0.01; Table 4).

Creatinine is correlated to oxidative stress marker. It has been previously reported that increased creatinine is correlated to increased glucose, which activates oxidative stress in patients with T2DM [24]. Terawaki et al. [25] and Huang et al. [26] reported that increased creatinine was significantly correlated to increased oxidative stress in patients with chronic kidney disease and end stage renal disease, respectively. In present study, significant correlation has been observed between creatinine and SPA and TOS and TAS and OSI in patients with T2DM and DN. In case of patients with ESRD, creatinine was significantly correlated to SPA, while nonsignificantly correlated to TOS, TAS, and OSI (Table 4).

After observing the correlation between serum glucose and creatinine with the oxidative markers, we evaluated these markers in reference to their role in the predictive value for kidney damage. In the present study SPA, TOS, TAS, and OSI sensitivity and specificity were observed for prediction of kidney damage. SPA value at ≥60.78 mmol Min^−1^ L^−1^ predicted 76% sensitivity with 66% of specificity for kidney damage (either DN or ESRD or both). TOS at ≥16.64 *μ*mol H_2_O_2_ Eq./L, TAS at ≤0.81 mmol Trolox Eq./L, and OSI at ≥10.24 were predicted 89.10% sensitivity with 60% specificity, 89.10% sensitivity with 2% specificity, and 80.60% sensitivity with 67% specificity for kidney damage (either DN or ESRD or both), respectively (Table 5 and Figure 1). It shows that increased values of SPA, TOS, and OSI and decreased TAS have more sensitivity rather than specificity for prediction of kidney damage. Thus it seems that increased SPA, TOS, and OSI and decreased TAS can be used as biomarkers for prediction of kidney damage.

This study revealed that serum prolidase activity and oxidative stress were significantly increased in patients with DN and ESRD than patients with T2DM and healthy volunteers. The same pattern was also obtained for T2DM with respect to healthy volunteers with significant differences. Blood glucose and creatinine were correlated to oxidative stress markers (TOS, TAS, and OSI) and serum prolidase activity in patients with T2DM, DN, and ESRD. It is concluded that serum prolidase activity, TOS, TAS, and OSI at cutoff values ≥60.78 mmol Min^−1^ L^−1^, ≥16.64 *μ*mol H_2_O_2_ Eq./L, ≤0.81 mmol Trolox Eq./L, and ≥10.24 AU, respectively, can be use as biomarkers for prediction and diagnosis of kidney damage (either DN or ESRD or both).

## Figures and Tables

**Table 1:** Representation of clinical parameters and demography for controls and cases.

Parameter	Control-1	Control-2	Case-1	Case-2	ANOVA (*F*/Sig.)
Number of subjects (total 229 subjects)	50	50	86	43	*⋯*
Sex (F = female, M = male)	F-20	F-16	F-39	F-19	*⋯*
	M-30	M-34	M-47	M-24	
Age mean (in years)	52.64 ± 18.76	53.34 ± 14.68	52.34 ± 12.89	57.34 ± 15.36	*F* = 1.16 *P* = 0.326
Duration of T2DM (in years)	Nil	7.18 ± 3.22	11.14 ± 3.18	16.46 ± 3.09	*F* = 100.45 *P*< 0.001
Fasting blood glucose (FBS), mg/dL	125.32 ± 43.91	191.26 ± 38.24	193.87 ± 42.27	218.91 ± 39.54	*F* = 46.01 *P*< 0.001
Postprandial blood glucose (PPBS), mg/dL	130.68 ± 47	236.68 ± 53.89	236.17 ± 23.79	255.84 ± 37.30	*F* = 102.58 *P*< 0.001
Serum creatinine, mg/dL	1.07 ± 0.34	1.91 ± 0.43	2.74 ± 0.60	6.05 ± 1.92	*F* = 241.73 *P*< 0.001
Glomerular filtration rate (GFR), mL/min/1.7 m^2^	86.13 ± 11.47	72.96 ± 16.14	41.59 ± 12.45	15.73 ± 6.31	*F* = 320.75 *P*< 0.001
Blood pressure (mm Hg)					
Systolic	127.52 ± 11.17	136.63 ± 7.85	138.94 ± 7.14	152.74 ± 9.47	*F* = 64.99 *P*< 0.001
Diastolic	81.50 ± 6.87	85.53 ± 7.63	87.53 ± 9.02	88.65 ± 7.79	*F* = 7.83 *P*< 0.001
Haemoglobin, g/dL	12.79 ± 1.39	11.56 ± 1.30	9.59 ± 1.71	8.86 ± 2.00	*F* = 63.66 *P*< 0.001
Blood urea, mg/dL	31.16 ± 14.24	49.66 ± 9.72	89.10 ± 23.40	128.98 ± 32.41	*F* = 192.86 *P*< 0.001

**Table 2:** Representation of SPA, TAS, TOS, and OSI for cases and controls.

Subjects/parameters	Control-1 (*n* = 50)	Control-2 (*n* = 50)	Case-1 (*n* = 86)	Case-2 (*n* = 43)	ANOVA (Sig./*F*)
SPA (mmol min^−1^ L^−1^)	55.72 ± 7.90 (*R* = 47.32 to 78.34)	60.18 ± 7.85 (*R* = 45 to 82)	68.56 ± 11.09 (*R* = 47.49 to 89.42)	74.23 ± 13.22 (*R* = 56 to 96)	*F* = 32.14 *P*< 0.001
TAS (mmol Trolox Eq/L)	1.97 ± 0.51 (*R* = 0.81 to 2.91)	1.82 ± 0.43 (*R* = 0.95 to 2.76)	1.42 ± 0.39 (*R* = 0.78 to 2.25)	1.20 ± 0.45 (*R* = 0.63 to 1.98)	*F* = 32.99 *P*< 0.001
TOS (*µ*mol H_2_O_2_ Eq/L)	13.82 ± 2.14 (*R* = 9.88 to 17.71)	17.54 ± 2.46 (*R* = 12.74 to 28.91)	20.13 ± 3.75 (*R* = 12.50 to 34.0)	22.21 ± 3.66 (*R* = 15.34 to 31.0)	*F* = 64.47 *P*< 0.001
OSI (arbitrary unit)	8.02 ± 4.21 (*R* = 3.41 to 21.86)	10.58 ± 4.58 (*R* = 5.04 to 29.50)	16.04 ± 7.58 (*R* = 5.55 to 42.50)	22.09 ± 10.21 (*R* = 7.87 to 47.69)	*F* = 37.21 *P*< 0.001

**Table 3:** Representation of Student-Newman-Keuls test's *P* value for different variable and multiple comparison groups.

Variables	*P* values for groups compared (according to Student-Newman-Keuls test)					
	Control-1 versus Control-2	Control-1 versus case-1	Control-1 versus case-2	Control-2 versus case-1	Control-2 versus Case-2	Case-1 versus Case-2
SPA	0.031	<0.001	<0.001	<0.001	<0.001	0.004
TAS	0.091	<0.001	<0.001	<0.001	<0.001	0.007
TOS	<0.001	<0.001	<0.001	<0.001	<0.001	0.001
OSI	0.07	<0.001	<0.001	<0.001	<0.001	<0.001
GFR	<0.001	<0.001	<0.001	<0.001	<0.001	<0.001
Serum Cr	<0.001	<0.001	<0.001	<0.001	<0.001	<0.001
FBS	<0.001	<0.001	<0.001	0.722	0.001	0.001
PPBS	<0.001	<0.001	<0.001	0.943	0.022	0.009
BP Systolic	<0.001	<0.001	<0.001	0.139	<0.001	<0.001
BP Diastolic	0.013	<0.001	<0.001	0.164	0.064	0.459
Hb	<0.001	<0.001	<0.001	<0.001	<0.001	0.016
B Urea	<0.001	<0.001	<0.001	<0.001	<0.001	<0.001

*P* values >0.05 considered as nonsignificant, while *P* values <0.05, <0.01, and <0.001 considered as significant, highly significant and very highly significant values, respectively.

**Table 4:** Representation of Pearson's correlation values for different comparison groups. FBS was nonsignificantly correlated to SPA, TOS, TAS, and OSI in control-1, while significantly correlated in control-2, case-1, and case-2. Nonsignificant correlation was observed between creatinine and SPA, TOS, TAS, and OSI in control-1, while significant in control-2 and case-1.

Correlative variables	Pearson's correlation values for controls		Pearson's correlation values for cases	
	Control-1	Control-2	Case-1	Case-2
	*r*-value	*r*-value	*r*-value	*r*-value
FBS Vs SPA	0.075^#^	0.801∗∗∗	0.674∗∗∗	0.820∗∗∗
FBS Vs TOS	0.025^#^	0.635∗∗∗	0.475∗∗∗	0.425∗∗
FBS Vs TAS	−0.074^#^	−0.612∗∗∗	−0.759∗∗∗	−0.580∗∗∗
FBS Vs OSI	0.030^#^	0.591∗∗∗	0.726∗∗∗	0.459∗∗
Cr Vs SPA	−0.024^#^	0.837∗∗∗	0.831∗∗∗	0.617∗∗∗
Cr Vs TOS	−0.066^#^	0.648∗∗∗	0.648∗∗∗	0.125^#^
Cr Vs TAS	−0.043^#^	−0.610∗∗∗	−0.718∗∗∗	−0.256^#^
Cr Vs OSI	0.002^#^	0.597∗∗∗	0.699∗∗∗	0.218^#^
DD Vs SPA	Nil	0.862∗∗∗	0.887∗∗∗	0.766∗∗∗
DD Vs TOS	Nil	0.441∗∗	0.591∗∗∗	0.324∗
DD Vs OSI	Nil	0.446∗∗	0.745∗∗∗	0.363∗
DD Vs TAS	Nil	−0.391∗∗	−0.859∗∗∗	−0.472∗∗

^#^Nonsignificant value; ∗significant value at *P*< 0.05; ∗∗significant value at *P*< 0.01; ∗∗∗significant value at *P*< 0.001; DD: duration of T2DM.

**Table 5:** Values of SPA, TOS, TAS, and OSI sensitivity and specificity for prediction of kidney damage (including both values of case-1 and case-2) with respect to control-1 and control-2.

Diagnosis	Cutoff level for kidney damage (DN or ESRD or both)	Sensitivity (%)	Specificity (%)	AUROC (95% CI)	Asymptotic Sig.
SPA (mmol min^−1^ L^−1^)	≥60.78	76	66	0.798 (0.741–0.855)	<0.001
TOS (*µ*mol H_2_O_2_ Eq/L)	≥16.64	89.10	60	0.87 (0.825–0.915)	<0.001
TAS (mmol Trolox Eq/L)	≤0.81	89.10	2	0.198 (0.141–0.255)	<0.001
OSI (arbitrary unit)	≥10.24	80.60	67	0.838 (0.787–0.889)	<0.001

AUROC: area under the receiver operating characteristic curve; CI: confidence interval.
